# Stochastic Dynamic Analysis and Vibration Suppression of FG-GPLRC Cylinder–Plate Combined Structures with Distributed Dynamic Vibration Absorbers

**DOI:** 10.3390/ma19061082

**Published:** 2026-03-11

**Authors:** Qingtao Gong, Ai Zhang, Yao Teng, Yuan Wang

**Affiliations:** 1 Ulsan Ship and Ocean College, Ludong University, Yantai 264025, China; 2 Shandong Marine Aerospace Technolgy Inovation Center, Yantai 264025, China; 3 Shandong Aerospace Harbor Research Institute, Yantai 265100, China; 4 Yantai Science and Technology Innovation Promotion Center, Yantai 264003, China

**Keywords:** cylinder-plate combined structures, functionally graded graphene platelet-reinforced composites, pseudo-excitation method, stochastic dynamics, vibration suppression

## Abstract

Cylinder–plate combined structures (CPCS) are widely used in aerospace, marine engineering, and offshore platform systems. During service, they are frequently subjected to stochastic excitations induced by turbulent boundary layers, acoustic loads, hydrodynamic disturbances, and broadband operational vibrations. Excessive random vibration responses may significantly reduce structural reliability, accelerate fatigue damage, and compromise operational safety. To address these engineering challenges, a unified stochastic dynamic analysis and vibration suppression framework is established for functionally graded graphene platelet-reinforced composites (FG-GPLRC) CPCS equipped with distributed dynamic vibration absorbers (DVAs). Adopting the First-order Shear Deformation Theory (FSDT), a comprehensive energy functional for the CPCS is established, in which the penalty method is implemented to impose boundary conditions and ensure interface continuity. Subsequently, the Pseudo-excitation Method (PEM) is utilized to convert the stochastic vibration analysis into an equivalent deterministic harmonic problem, and the governing equations are spatially discretized by combining the spectral geometric method (SGM) with the Ritz variational procedure, enabling efficient evaluation of power spectral density (PSD) and root-mean-square (RMS) responses. The reliability of the proposed model is verified through a series of numerical validation comparisons. On this basis, comprehensive parametric investigations are conducted to assess how material properties, structural geometries, and critical DVA parameters influence system behavior. The results demonstrate that the incorporation of distributed DVAs can achieve superior vibration suppression performance. This study provides an efficient and reliable theoretical framework for stochastic vibration analysis and damping design of advanced composite plate–shell coupled structures operating in complex random environments, offering important theoretical support for dynamic optimization design in aerospace and marine engineering applications.

## 1. Introduction

Plate–shell combined structures deployed in aerospace, marine, and high-end mechanical systems are continuously exposed to complex stochastic environments during service, such as turbulent boundary-layer fluctuations, acoustic loads, hydrodynamic pressure variations, and broadband mechanical disturbances. Under such random excitations, excessive vibration responses may significantly reduce structural reliability, accelerate fatigue damage, and compromise operational safety. Owing to their high stiffness-to-weight ratio and superior load-transfer capability, cylinder–plate combined structures (CPCS) are widely adopted in engineering applications where curved shells and flat plates must function integrally, including cabin-like aerospace components, underwater pressure-resistant assemblies, and launch-support structures. The cylinder–plate configuration is particularly representative as it captures the essential coupling characteristics between curved and flat substructures, making it an ideal benchmark for understanding the dynamic behavior of more complex assembled systems. Meanwhile, functionally graded graphene platelet-reinforced composites (FG-GPLRC) have emerged as promising advanced materials due to their tailorable stiffness distribution, enhanced mechanical performance, and adaptability to severe service conditions. However, when CPCS fabricated from FG-GPLRC operate under broadband stochastic excitation, the coupling effects between substructures, material gradation, and dynamic interactions may lead to complex vibration characteristics that are not yet fully understood. Therefore, systematically investigating the stochastic dynamic behavior of FG-GPLRC CPCS and exploring effective vibration suppression strategies is of considerable engineering importance and theoretical significance.

In recent years, extensive research efforts have been devoted to the mechanical modeling and dynamic analysis of FG-GPLRC structures. Owing to the exceptional stiffness and strength of graphene platelets, FG-GPLRC materials exhibit significantly enhanced structural performance compared with conventional fiber-reinforced composites. Driven by these merits, considerable scholarly attention has been directed toward analyzing the dynamic behavior of FG-GPLRC components across a spectrum of mechanical and environmental settings. Furthermore, to bolster the fidelity of structural simulations, advanced plate–shell theories are frequently utilized. For example, high-order shear deformation theory (HSDT) has been employed to analyze the dynamic response of FG-GPLRC annular plates subjected to thermal environments and elastic foundations [1], while First-order Shear Deformation Theory (FSDT) has been extensively applied to examine buckling and vibration responses of cylindrical shells and annular plates [2,3,4]. In addition, Reddy’s third-order shear deformation theory has been used to investigate nonlinear vibration characteristics of curved shell structures [5], and Timoshenko beam theory has been introduced to study coupled vibration behavior in disk–shell systems [6]. From a more refined perspective, three-dimensional elasticity formulations have also been adopted to enhance prediction accuracy for FG-GPLRC shell vibrations [7,8]. To address geometric nonlinearity and complex deformation effects, von Kármán-type nonlinear assumptions have frequently been incorporated into the governing equations [9], and Donnell’s shell kinematic relations have been utilized for cylindrical shell modeling [10]. In terms of solution methodologies, Hamilton’s principle has commonly served as the fundamental variational framework [7], with numerical implementations realized through generalized differential quadrature methods (GDQM) [1], generalized differential quadrature element (GDQE) methods [11], Galerkin approaches, enhanced Ritz formulations [12], and FEM. Through these modeling and computational strategies, researchers have systematically explored the influences of graphene platelet (GPL) distribution patterns and volume fractions [3], geometric parameters [13], nonlinear elastic foundations [14], thermal environments [1], and rotational motion on natural frequencies, buckling loads, nonlinear vibration amplitudes, and critical rotational speeds of FG-GPLRC structures.

Combined plate-shell structures are widely employed in practical engineering due to their ability to integrate the load-bearing advantages of curved shells and flat plates within a unified configuration. Their dynamic characteristics have therefore attracted sustained research interest. Early investigations focused on clarifying the fundamental vibration behavior of shell–plate assemblies, where extended Rayleigh–Ritz formulations were introduced to evaluate the free vibration characteristics of cylindrical shells coupled with internal plates and to assess the influence of connection stiffness on system frequencies [15]. Subsequent studies further examined coupling mechanisms from different physical perspectives. For instance, wavenumber spectrum approaches were developed to examine the acoustic interaction performance of cylindrical shells fitted with internal floor partitions, revealing the interaction between structural vibration and acoustic fields [16]. From the viewpoint of energy transmission, substructure-based methods were proposed to explore the energy transmission behavior of plate–cylindrical shell integrated systems, providing insight into vibration energy transfer within complex assemblies [17]. To improve modeling generality and computational efficiency, dynamic stiffness matrix formulations were constructed for open cylindrical shell–plate interconnected configurations using generalized superposition and projection techniques, extending FEM-like concepts within the dynamic stiffness framework [18]. In parallel, unified theoretical modeling strategies have been developed to systematically treat boundary conditions and coupling relationships in plate–shell systems [19]. By combining improved Fourier series expansions with Ritz-type procedures, researchers established generalized vibration models for cylindrical shell–ring plate coupling structures and examined the effects of elastic coupling conditions on dynamic responses [20]. Similar unified formulations were later extended to open cylindrical shell–rectangular plate systems, enabling parametric analyses of how geometric and coupling parameters influence natural frequencies and modal characteristics [1].

Given the intricate operational settings prevalent in aerospace, marine, and mechanical engineering, structural assemblies are frequently exposed to stationary or nonstationary stochastic excitations. This reality has prompted widespread scholarly interest in analyzing structural random vibration behavior [21,22,23,24]. Early investigations mainly focused on laminated and thin plate structures, where FSDT was employed to analyze dynamic responses under nonstationary random excitation [25]. Analytical methodologies were also devised to assess the stochastic vibration properties of rectangular thin plates, elucidating the impact of boundary constraints and excitation frequency bands on the response statistics [26]. Finite element-based frameworks were further established to study structural responses under combined stochastic and convective loads, enhancing modeling flexibility for complex loading environments [27]. In addition, Galerkin-type procedures were utilized to derive the stochastic responses of plates supported by elastic foundations, elucidating the impact of excitation magnitude and foundation properties on the dynamic behavior [28]. To improve computational efficiency in stochastic dynamic analysis, the Pseudo-excitation Method (PEM) was introduced as an effective alternative to traditional spectral or Monte Carlo approaches, enabling random vibration problems to be transformed into equivalent deterministic harmonic analyses [29]. Based on PEM theory, structural–acoustic coupled models were constructed to investigate sound pressure spectral densities and acoustic sensitivities under random excitation [30], and wavelet-based extensions were further proposed to analyze beam–soil systems subjected to moving stochastic loads [31]. Integrating the PEM with the Improved Fourier Series Method (IFSM) facilitated the efficient examination of both stationary and nonstationary stochastic oscillations in laminated plates subjected to combined thermal and aerodynamic loading. This approach underscored the critical impact of thermal environments, material attributes, and aerodynamic pressures [32]. Moreover, discrete analytical methods (DAM) integrated with PEM were developed to enhance computational accuracy and efficiency, with validation against FEM results [33], and fully nonstationary random dynamic models were subsequently established to capture the influence of time-varying stochastic excitations on plate behavior [34].

As typical passive damping devices, dynamic vibration absorbers (DVAs) have been extensively applied in engineering structures owing to their ability to effectively reduce structural vibrations without requiring external energy input. Research has shown that, through rational design and optimization of DVAs stiffness, mass, and tuned frequency, DVAs can effectively attenuate resonant responses under specific excitations and enhance the dynamic stability of structural systems. Based on these capabilities, extensive studies have been conducted to develop DVA-based vibration suppression strategies for various structural forms and loading conditions [35]. In the context of plates and shells, researchers have employed analytical, numerical, and semi-analytical approaches to systematically investigate the dynamic response of structures equipped with distributed DVAs [36]. These studies indicate that the selection and configuration of DVA parameters, such as mass distribution, placement, and tuning frequency, have significant influence on forced vibration amplitudes, frequency response characteristics, and energy transfer mechanisms. Moreover, distributed DVAs have been demonstrated to offer superior broadband vibration control compared to single-point absorbers in many cases [37,38]. Recent research has also extended the application of DVAs to more complex structural forms, such as thin-walled panels integrated with acoustic black hole (ABH) treatments, where the design and placement of distributed absorbers further enhance broadband attenuation performance [39].

Although substantial progress has been achieved in the dynamic modeling of functionally graded graphene-reinforced composite structures, the coupled vibration analysis of plate–shell systems, and stochastic vibration analysis methods, systematic investigations on the random vibration behavior and vibration control of functionally graded graphene-reinforced plate–shell coupled structures equipped with dynamic vibration absorbers remain relatively limited. Therefore, this study focuses on FG-GPLRC CPCS with distributed dynamic vibration absorbers and systematically investigates its stochastic dynamic characteristics under random excitation. A unified dynamic model of the FG-GPLRC plate–shell coupled structure with DVAs is established, and an efficient random vibration analysis framework integrating the pseudo-excitation method is developed to evaluate the stochastic response of the system. Parametric studies are further conducted to reveal the influences of absorber parameters, material gradation patterns, and structural characteristics on the vibration suppression performance. The results provide theoretical guidance for the vibration control design of advanced composite plate–shell structures operating in complex stochastic environments.

## 2. Theoretical Modeling

### 2.1. Geometric Description of Structural Models

**Figure 1:**
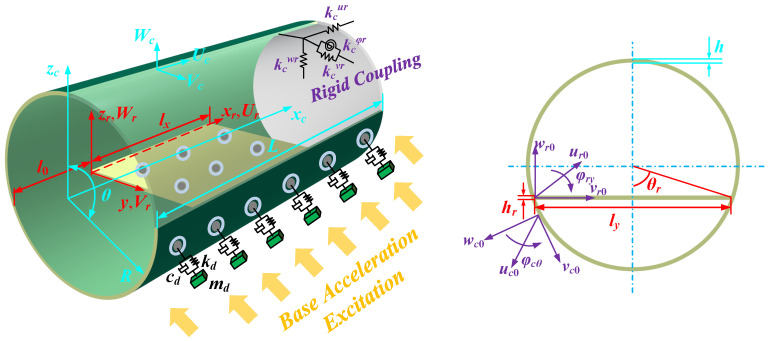
Schematic illustration of the geometric configuration of the CPCS-DD.

The schematic of the CPCS equipped with distributed DVAs (CPCS-DD) configuration, including its geometric dimensions and coordinate systems, is shown in Figure 1. The CPCS is formed by rigidly integrating a cylindrical shell with a rectangular plate, and the distributed DVAs are evenly mounted on the exterior surface of the cylindrical shell. The geometric parameters of the cylindrical shell and the rectangular plate are (*L*, *R*, *h*) and (*l_x_*, *l_y_*, *h_r_*), respectively. A Cartesian coordinate system (*o*-*αβz_q_*) is defined on the mid-surface of each substructure, where the subscript *q* = *c*, *r* denotes the cylindrical shell and the rectangular plate, respectively. The *α* and *β* align with the meridional and circumferential directions. For the cylindrical shell, *α* = *x_c_* and *β* = *θ*; for the rectangular plate, *α* = *x_r_* and *β* = *y*. The displacement components of each substructure in the orthogonal coordinate system are denoted by (*U_q_*, *V_q_*, *W_q_*). The position of the rectangular plate is determined by *l*_0_ and the included angle *θ_r_*, yielding *l_y_* = 2*R*sin*θ_r_*. Each DVA is idealized as a mass–spring–damper assembly with parameters (*m_d_*, *k_d_*, *c_d_*). To simulate boundary constraints on the structure, rigid springs ($k_{c}^{ur}$, $k_{c}^{vr}$, $k_{c}^{wr}$, $k_{c}^{\theta r}$) were employed to model the rigid coupling between the cylindrical shell and rectangular plate. The stiffness value of the coupling springs was set to 10 × 10^14^. The plate and shell are also rigidly connected at the interface.

### 2.2. Modeling of Stochastic Excitation

Grounded in the theoretical principles of the PEM, the stochastic excitation *g* considered herein is formulated as

(1) $$g\left(\alpha ,\beta ,t\right)=-I_{i}\sqrt{G_{g}}e^{i\omega t}$$

where *I_i_* denotes the mass moment of inertia, whose explicit expression can be found in Ref. [40], and Gg represents the stochastic excitation. The CPCS is subjected to excitation along the *z*-direction of the global coordinate frame. Owing to the distinct geometric features of the rectangular plate and the cylindrical shell, the relationships between their local coordinate systems and the global coordinate system differ accordingly. Therefore, the virtual excitation expressions corresponding to the displacement components of each substructure are different. The specific formulations are given as follows:

(2) $$\left\{\begin{array}{l} g_{u}^{c}\left(\alpha ,\beta ,t\right)=0\\ g_{v}^{c}\left(\alpha ,\beta ,t\right)=g\left(\alpha ,\beta ,t\right)\cos\beta \\ g_{w}^{c}\left(\alpha ,\beta ,t\right)=g\left(\alpha ,\beta ,t\right)\sin\beta \end{array}\right.\left\{\begin{array}{l} g_{u}^{r}\left(\alpha ,\beta ,t\right)=0\\ g_{v}^{r}\left(\alpha ,\beta ,t\right)=0\\ g_{w}^{r}\left(\alpha ,\beta ,t\right)=g\left(\alpha ,\beta ,t\right) \end{array}\right.$$

where the superscripts *c* and *r* denote the excitations applied to the cylindrical shell and the rectangular plate, respectively.

### 2.3. Characterization of FG-GPLRC

In the present work, the CPCS is composed of FG-GPLRC. The material-design rationale of FG-GPLRC is that GPLs are dispersed within a polymer matrix according to a prescribed rule; consequently, every individual layer is modeled as an isotropic medium, whereas the GPL mass fraction exhibits a gradient distribution along the thickness direction of the plate–shell assembly. The effective Young’s modulus of the *k*-th layer can be predicted using the Halpin–Tsai micromechanical model as follows:

(3) $$E^{k}=\frac{3\left(1+\gamma_{L}\varsigma_{L}V_{G}^{k}\right)}{8\left(1-\varsigma_{L}V_{G}^{k}\right)}E_{M}+\frac{5\left(1+\gamma_{W}\varsigma_{W}V_{G}^{k}\right)}{8\left(1-\varsigma_{W}V_{G}^{k}\right)}E_{M}$$

where *E_M_* is the Young’s modulus of the polymer matrix, and $V_{G}^{k}$ represents the GPL volume fraction within the *k*-th layer. *γ_L_*, *γ_W_*, $\varsigma_{L}$, and $\varsigma_{W}$ are intrinsic material parameters that can be determined from the GPL Young’s modulus *E_G_*, *E_M_*, and the GPL geometry, namely the length *l_G_*, width *w_G_*, and thickness *h_G_*; the detailed expressions are referred to Ref. [40]. The expression of $V_{G}^{k}$ can be evaluated from the mass fraction of GPLs, *g^k^* within each layer, the GPL density *ρ_G_*, and the polymer-matrix density *ρ_M_* according to the following relation:

(4) $$V_{G}^{k}=\frac{g^{k}}{g^{k}+\left(\rho_{G}/\rho_{M}\right)\left(1-g^{k}\right)}$$

Under different distribution patterns, the mass fraction of GPLs present in the *k*-th layer of the FG-GPLRC varies accordingly. In this study, four representative distribution types are considered. These distribution patterns represent different through-thickness dispersion characteristics of GPLs in the FG-GPLRC: the G-U pattern denotes a uniform distribution throughout the thickness, the G-O pattern indicates that GPLs are mainly concentrated near the mid-plane of the structure, the G-X pattern represents GPL enrichment near the top and bottom surfaces, while the G-Λ pattern corresponds to a linear gradient distribution along the thickness direction. The corresponding expressions of *g^k^* are given as follows:

(5) $$g^{k}=\left\{\begin{array}{ll} g^{T} & G-U\\ 2g^{T}\frac{N_{F}+1-\left|2k-N_{F}-1\right|}{N_{F}+2} & G-O\\ 2g^{T}\frac{1+\left|2k-N_{F}-1\right|}{N_{F}+2} & G-X\\ 2g^{T}\frac{k}{N_{F}+1} & G-\Lambda \end{array}\right.$$

where *g^T^* denotes the total GPL mass fraction, and *N_F_* is the total number of layers in the FG-GPLRC. The effective density and effective Poisson’s ratio of the *k*-th layer can be obtained based on the rule of mixtures as

(6) $$\begin{array}{l} \mu^{k}=\mu_{G}V_{G}^{k}+\mu_{M}\left(1-V_{G}^{k}\right)\\ \rho^{k}=\rho_{G}V_{G}^{k}+\rho_{M}\left(1-V_{G}^{k}\right) \end{array}$$

where *μ_G_* and *μ_M_* represent the Poisson’s ratios corresponding to the GPL and the polymer matrix, respectively.

### 2.4. Energy Functional Formulation of the System

Within the framework of the FSDT, the displacement-field vector of each substructure in the CPCS, ***D_q_*** = [*U_q_*, *V_q_*, *W_q_*]^T^, can be expressed in terms of the mid-surface displacement components ***M_q_*** = [*u_q_*_0_, *v_q_*_0_, *w_q_*_0_, *φ_q__α_*, *φ_qβ_*]^T^. The corresponding relations are given as follows:

(7) $$D_{q}=Q_{m}M_{q}$$

where ***Q_m_*** is the coefficient transformation matrix, whose explicit form is provided in Ref. [41].

Within the framework of elasticity theory, the strain–displacement relations at an arbitrary point of each substructure in the CPCS can be written as follows [42]:

(8) $$\begin{array}{l} \varepsilon_{\alpha }^{k}=\frac{1}{AB}\left(\frac{B\partial u_{q}}{\partial \alpha }+\frac{v_{q}\partial A}{\partial \beta }+\frac{ABw_{q}}{R_{\alpha }}+\frac{Bz_{q}\partial \varphi_{q\alpha }}{\partial \alpha }+\frac{z_{q}\varphi_{q\beta }\partial A}{\partial \beta }\right)\\ \varepsilon_{\beta }^{k}=\frac{1}{AB}\left(\frac{A\partial v_{q}}{\partial \beta }+\frac{u_{q}\partial B}{\partial \alpha }+\frac{ABw_{q}}{R_{\beta }}+\frac{z_{q}A\partial \varphi_{q\beta }}{\partial \beta }+\frac{z_{q}\varphi_{q\alpha }\partial B}{\partial \alpha }\right)\\ \gamma_{\alpha \beta }^{k}=\frac{1}{AB}\left(\frac{B\partial v_{q}}{\partial \alpha }+\frac{A\partial u_{q}}{\partial \beta }-\frac{u_{q}\partial A}{\partial \beta }-\frac{v_{q}\partial B}{\partial \alpha }+\frac{z_{q}B\partial \varphi_{q\beta }}{\partial \alpha }+\frac{z_{q}A\partial \varphi_{q\alpha }}{\partial \beta }-\frac{z_{q}\varphi_{q\beta }\partial A}{\partial \beta }-\frac{z_{q}\varphi_{q\alpha }\partial B}{\partial \alpha }\right)\\ \gamma_{\alpha z_{q}}^{k}=\frac{1}{A}\left(A\varphi_{q\alpha }-\frac{Au_{q}}{R_{\alpha }}+\frac{\partial w_{q}}{\partial \alpha }\right)\\ \;\gamma_{\beta z_{q}}^{k}=\frac{1}{B}\left(A\varphi_{q\beta }-\frac{Av_{q}}{R_{\beta }}+\frac{\partial w_{q}}{\partial \beta }\right) \end{array}$$

where *A* and *B* are the Lamé parameters, and *R_α_* and *R_β_* denote the principal radii of curvature. Their explicit expressions can be found in Refs. [43,44].

Under the assumption of linear-elastic deformation of the plate–shell structure, the stress–strain constitutive relations for the *k*-th layer can be derived from Hooke’s law as

(9) $$Y^{k}=Q_{y}B^{k}$$

where ***Y^k^*** = [$\sigma_{\alpha }^{k}$, $\sigma_{\beta }^{k}$, $\tau_{\alpha \beta }^{k}$, $\tau_{\alpha z_{q}}^{k}$, $\tau_{\beta z_{q}}^{k}$]^T^ is the stress vector, ***B^k^*** = [$\varepsilon_{\alpha }^{k}$, $\varepsilon_{\beta }^{k}$, $\gamma_{\alpha \beta }^{k}$, $\gamma_{\alpha z_{q}}^{k}$,$\gamma_{\beta z_{q}}^{k}$]^T^, and ***Q_y_*** is the material stiffness correction coefficient, whose explicit expression can be found in Ref. [45].

By applying the kinetic energy theorem, the kinetic energy of each substructure of the CPCS can be expressed as

(10) $$T_{q}=\frac{1}{2}\int_{V_{q}}\rho^{k}{\left({\;\overset{\cdot }{D}}_{q}\right)}^{T}{\;\overset{\cdot }{D}}_{q}dV_{q}$$

Based on elasticity theory, the strain energy of each substructure of the CPCS can be expressed as

(11) $$U_{q}=\frac{1}{2}\iint {\left(Y^{k}\right)}^{T}B^{k}d\alpha d\beta$$

Because the cylindrical shell and the rectangular plate in the CPCS are coupled through an interface connection, the potential energy associated with interfacial coupling at the connection is formulated using the penalty-parameter method as

(12) $$P^{c}=\frac{1}{2}\iint {\left.s_{1}^{T}K_{c}s_{1}\right|}_{y=0,\theta =\frac{\pi }{2}-\theta_{r}}+{\left.s_{2}^{T}K_{c}s_{2}\right|}_{y=b,\theta =\frac{\pi }{2}+\theta_{r}}dx_{r}dz_{r}$$

where ***K_c_*** = diag ($k_{c}^{u_{r}}$, $k_{c}^{v_{r}}$, $k_{c}^{w_{r}}$, $k_{c}^{\varphi_{ry}}$) denotes the coupling spring stiffness matrix, and ***s*_1_** and ***s*_2_** represent the relative displacement components at the coupling boundary. Their specific expressions are given as follows:

(13) $$\begin{array}{l} s_{1}={\left[\begin{array}{cccc} u_{r}-u_{c} & v_{r}+w_{c}\sin\theta_{r}-v_{c}\cos\theta_{r} & w_{r}-v_{c}\sin\theta_{r}-w_{c}\cos\theta_{r} & \varphi_{ry}-\varphi_{c\theta } \end{array}\right]}^{T}\\ s_{2}={\left[\begin{array}{cccc} u_{r}-u_{c} & v_{r}-w_{c}\sin\theta_{r}-v_{c}\cos\theta_{r} & w_{r}-w_{c}\cos\theta_{r}+v_{c}\sin\theta_{r} & \varphi_{ry}-\varphi_{c\theta } \end{array}\right]}^{T} \end{array}$$

Similarly, the penalty-parameter method is employed to provide an equivalent representation of the boundary constraints of the CPCS, and the corresponding boundary potential energy is expressed as

(14) $$\begin{array}{ll} P^{b}= & \frac{1}{2}\iint {\left.M_{c}^{T}K_{b}^{c0}M_{c}\right|}_{x_{c}=0}+{\left.M_{c}^{T}K_{b}^{c1}M_{c}\right|}_{x_{c}=L}d\theta dz_{c}+\\ & \frac{1}{2}\iint {\left.M_{r}^{T}K_{b}^{r0}M_{r}\right|}_{x_{r}=l_{0}}+{\left.M_{r}^{T}K_{b}^{r1}M_{r}\right|}_{x_{r}=l_{0}+l_{x}}d\theta dz_{c} \end{array}$$

where $K_{b}^{\mathrm{qo}}$ = diag ($k_{b}^{u_{q}ο}$, $k_{b}^{v_{q}ο}$, $k_{b}^{w_{q}ο}$, $k_{b}^{\varphi_{q\alpha }ο}$, $k_{b}^{\varphi_{q\beta }ο}$) (*ο* = 0, 1) denotes the penalty parameters associated with different boundaries, which are represented in the form of equivalent spring stiffnesses.

Analogous to the kinetic energy expression of the CPCS substructures, the kinetic energy of the DVA can be written as

(15) $$T_{de}=\frac{1}{2}m_{d}{\dot{h}}_{e}^{2}$$

where *h_e_* denotes the displacement of the *e*-th DVA in the *z_c_* direction at its installation location.

Analogous to the strain energy expression of the CPCS substructures, the strain energy of the DVA can be expressed as

(16) $$U_{de}=\frac{1}{2}k_{d}{\left(W_{c}^{e}-h_{e}\right)}^{2}$$

where $W_{c}^{e}$ denotes the displacement of the cylindrical shell in the *z_c_* direction at the installation location of the *e*-th DVA. The coordinate representation of the installation position of the *e*-th DVA is given as

(17) $$\begin{array}{l} x_{e}=x_{n_{\beta }\left(j_{\alpha }-1\right)+j_{\beta }}=\frac{j_{\alpha }}{n_{\alpha }+1}L\\ \theta_{e}=\theta_{n_{\beta }\left(j_{\alpha }-1\right)+j_{\beta }}=-\frac{\pi }{2}+\frac{2\pi }{j_{\beta }}\left(j_{\beta }-1\right) \end{array},j_{\alpha }=1,2,\ldots ,n_{\alpha },j_{\beta }=1,2,\ldots ,n_{\beta }$$

where *n_α_* and *n_β_* denote the numbers representing the quantity of DVAs distributed along the axial and circumferential directions, respectively.

The damping dissipation work of the *e*-th DVA is expressed as

(18) $$W_{de}^{l}=\frac{1}{2}c_{d}{\left({\dot{W}}_{c}^{e}-{\dot{h}}_{e}\right)}^{2}$$

Utilizing the functional relationship, the work performed by the stochastic excitation exerted on the surfaces of the CPCS substructures is formulated as

(19) $$W_{s}^{q}=\iint \left(g_{u}^{q}u_{q}+g_{v}^{q}v_{q}+g_{w}^{q}w_{q}\right)ABd\beta d\alpha$$

On this basis, the Lagrangian energy functional of the CPCS-DD system can be obtained as

(20) $$J=T_{c}+T_{r}+\sum_{e=1}^{N_{T}}T_{de}-U_{c}-U_{r}-\sum_{e=1}^{N_{T}}U_{de}-P^{b}-P^{c}+\sum_{e=1}^{N_{T}}W_{de}^{l}+W_{s}^{c}+W_{s}^{r}$$

### 2.5. Theoretical Formulation of the SGM Solution Method

When solving the governing dynamic problem, selecting appropriate admissible displacement functions is crucial for both the accuracy and computational efficiency of the solution. In this study, the SGM is employed to represent the structural displacement fields, and the corresponding expressions are given as follows:

(21) $$f\left(\alpha ,\beta ,t\right)=P_{\alpha }\left(\alpha \right)⊗P_{\beta }\left(\beta \right)Ae^{i\omega t}$$

where **P*_α_*** (*α*) and **P*_β_*** (*β*) are spectral geometric series, and **A** is the vector of unknown displacement coefficients. To characterize the expansion of the axial displacement for the cylindrical shell and the associated displacement field of the rectangular plate, the expressions of **P*_α_*** (*α*) and **P*_β_*** (*β*) are given as follows:

(22) $$\begin{array}{l} P_{\alpha }\left(\alpha \right)=\left[y_{-2}\left(\alpha \right),y_{-1}\left(\alpha \right),y_{0}\left(\alpha \right),y_{1}\left(\alpha \right),y_{2}\left(\alpha \right),\ldots ,y_{m}\left(\alpha \right),\ldots ,y_{M_{\alpha }}\left(\alpha \right)\right]\\ P_{\beta }\left(\beta \right)=\left[y_{-2}\left(\beta \right),y_{-1}\left(\beta \right),y_{0}\left(\beta \right),y_{1}\left(\beta \right),y_{2}\left(\beta \right),\ldots ,y_{n}\left(\beta \right),\ldots ,y_{N_{\beta }}\left(\beta \right)\right] \end{array}$$

where *M_α_* and *N_β_* denote the truncation numbers of the admissible functions, and *y_z_* (*t*) (*z* = *m*, *n*; *t* = *α*, *β*) represents the *z*-th term of the spectral geometric series, defined as

(23) $$y_{z}\left(t\right)=\left\{\begin{array}{c} \begin{array}{cc} \sin\left(\frac{z\pi t}{l}\right) & z<0 \end{array}\\ \begin{array}{cc} \cos\left(\frac{z\pi t}{l}\right) & z\geq 0 \end{array} \end{array}\right.$$

where *l* denotes the length in the *t*-direction.

For the circumferential displacement expansion of the cylindrical shell, a circumferential wave-number representation is adopted, i.e., **P*_θ_*** (*θ*) = [1, cos (*θ*), cos (2*θ*),…, cos (*N_θ_θ*), 0, sin (*θ*), sin (2*θ*),…, sin (*N_θ_θ*)], where *N_θ_* is the total circumferential wave number.

Therefore, the displacement components of each substructure in the CPCS along the corresponding directions can be expanded as follows:

(24) $$\begin{array}{l} u_{q0}=P_{\alpha }\left(\alpha \right)⊗P_{\beta }\left(\beta \right)A_{u}^{q}e^{i\omega t}\\ v_{q0}=P_{\alpha }\left(\alpha \right)⊗P_{\beta }\left(\beta \right)A_{v}^{q}e^{i\omega t}\\ w_{q0}=P_{\alpha }\left(\alpha \right)⊗P_{\beta }\left(\beta \right)A_{w}^{q}e^{i\omega t}\\ \varphi_{q\alpha }=P_{\alpha }\left(\alpha \right)⊗P_{\beta }\left(\beta \right)A_{\varphi_{a}}^{q}e^{i\omega t}\\ \varphi_{q\beta }=P_{\alpha }\left(\alpha \right)⊗P_{\beta }\left(\beta \right)A_{\varphi_{\beta }}^{q}e^{i\omega t} \end{array}$$

In a similar manner, the displacement expansion corresponding to the *e*-th DVA is formulated as

(25) $$h_{e}\left(x_{e},\theta_{e},t\right)=H_{e}e^{i\omega t}$$

Upon inserting the admissible displacement functions into Equation (20) and implementing the Ritz variational procedure, the spatially discretized governing equation for the CPCS–DD system is derived as

(26) $$M\ddot{\nu }+C\dot{\nu }+K\nu =F$$

where **M**, **C**, **K**, and **F** denote the mass matrix, damping matrix, stiffness matrix, and external load vector, respectively, and **ν** signifies the global vector of unknown displacement coefficients associated with the CPCS-DD system.

In stochastic dynamic analysis, the random response is expressed in terms of *w_q_*_0_ and its complex conjugate ${\tilde{w}}_{q0}$ as

(27) $$\begin{array}{ll} P_{g}\left(\alpha ,\beta ,t\right)={\tilde{w}}_{q0}\left(\alpha ,\beta ,t\right)w_{q0}\left(\alpha ,\beta ,t\right) & Dis._{PSD}\\ P_{\dot{g}}\left(\alpha ,\beta ,t\right)=\omega^{2}P_{g}\left(\alpha ,\beta ,t\right) & Vel._{PSD}\\ P_{\ddot{g}}\left(\alpha ,\beta ,t\right)=\omega^{4}P_{g}\left(\alpha ,\beta ,t\right) & Acc._{PSD} \end{array}$$

The RMS of the stochastic response is calculated as

(28) $$\begin{array}{ll} \sigma_{g}=\sqrt{\int_{\omega_{1}}^{\omega_{2}}\left|P_{g}\right|d\omega } & Dis._{RMS}\\ \sigma_{\dot{g}}=\sqrt{\int_{\omega_{1}}^{\omega_{2}}\left|P_{\dot{g}}\right|d\omega } & Vel._{RMS}\\ \sigma_{\ddot{g}}=\sqrt{\int_{\omega_{1}}^{\omega_{2}}\left|P_{\ddot{g}}\right|d\omega } & Acc._{RMS} \end{array}$$

where *ω*_1_ and *ω*_2_ denote the lower and upper cutoff frequencies in the stochastic dynamic analysis, respectively.

## 3. Numerical Investigation and Discussion

This section focuses on verifying the convergence behavior and numerical precision of the developed approach by means of multiple numerical case studies, which are based on the established dynamic model. On this basis, a comprehensive parametric study is further conducted to the dynamic characteristics of the coupled plate–shell system subjected to different parameter configurations, with particular emphasis on the effects of parameter variations and the installation of dynamic vibration absorbers (DVAs) on the coupled structural responses. Unless explicitly stated otherwise, all parameters of the coupled system remain unchanged across the numerical analyses. The dimensions of the cylindrical shell are specified as *L* = 6 m, *R* = 1 m, *h* = 0.1 m. The dimensions of the rectangular plate are given by *l_x_* = 6 m, *θ_r_* = 60°, *h_r_* = 0.1 m. The cylindrical shell and the rectangular plate are assumed to be composed of the same material, with the following material properties: *g^T^* = 0.01, *l_G_* = 2.5 μm, *w_G_* = 2.5 μm, *h_G_* = 1.5 nm, *N_F_* = 10, *E_G_* = 1010 GPa, *E_M_* = 3 GPa, *μ_G_* = 0.186, *μ_M_* = 0.34, *ρ_G_* = 1060 kg/m^3^, *ρ_M_* = 1200 kg/m^3^, *m_d_* = 2.3 kg, *k_d_* (G-UD) *=* 922,236 N/m, *k_d_* (G-O) *=* 791,915 N/m, *k_d_* (G-X) *=* 1,013,610 N/m, *k_d_* (G-Λ) = 849,938 N/m, *n_α_* = 5, *n_β_* = 5, *G_g_* = 1 g^2^/Hz. The frequency range of the stochastic excitation is specified as [20, 1000] Hz. The coordinates of response point 1# are (0.5 L, 90°), while those of response point 2# are (0.5 (*l*_0_ + *l_x_*), 0.5 *l_y_*). Clamped boundary conditions (C) are considered in this study, and the boundary conditions are denoted using string representations for clarity, such as “CC-CC”. For the convergence and validation analyses, the finite element outcomes derived via the commercial package Abaqus serve as the benchmark, in which S4R shell elements are employed for the FEM discretization.

### 3.1. Model Validation

The selection of truncation numbers has a direct influence on both the accuracy of the numerical results and the computational efficiency. Elevating the truncation numbers typically yields superior solution accuracy; nevertheless, it simultaneously induces a substantial surge in computational cost, which can exhibit exponential growth characteristics. Therefore, it is essential to perform a convergence analysis to determine appropriate truncation numbers for the proposed formulation. Table 1 reports the natural frequencies of the G-X type CPCS with clamped boundary conditions obtained using different truncation numbers. Corresponding to the displacement components of the rectangular plate in the *x*- and *y*-directions are the truncation numbers *M_xr_* and *N_y_*, respectively; the axial displacement component of the cylindrical shell, meanwhile, is associated with the truncation number *M_xc_*.

As illustrated in Table 1, once *M_xr_* and *N_y_* reach or exceed 15, and *M_xc_* is set to at least 16, the calculated natural frequencies remain essentially unchanged with further increases in truncation numbers, indicating that convergence has been achieved. Based on this observation, the truncation numbers are set to *M_xr_* = *N_y_* = 20 and *M_xc_* = 20 in the present study to ensure a satisfactory balance between computational accuracy and efficiency. This truncation configuration is adopted in all subsequent numerical investigations. To further validate the accuracy of the proposed theoretical model, finite element simulations were carried out and used for comparison. The FEM model of the cylindrical shell–rectangular plate coupled structure was established using commercial finite element software ABAQUS2025. Both the cylindrical shell and rectangular plate were modeled using shell elements and discretized with free quadrilateral meshes, yielding a total of 120,000 mesh elements, and the material properties of the FG-GPLRC were implemented through a layer-wise definition. The boundary conditions applied in the FEM simulations were kept consistent with those used in the theoretical formulation. A sufficiently refined mesh was employed to ensure the convergence and reliability of the numerical results.

To verify the accuracy and reliability of the dynamic model developed in this study, representative benchmark examples reported in the existing literature are selected for comparison, and the natural frequencies of FG-GPLRC cylindrical shells and rectangular plates are systematically examined. Table 2 presents a comparison of the first eight natural frequencies of FG-GPLRC cylindrical shells with four different GPL distribution patterns. It can be observed that, for all distribution types and modal orders, the results obtained by the present method show excellent agreement with the reference solutions, demonstrating the high accuracy of the proposed model in predicting the natural frequencies of cylindrical shells. Furthermore, Table 3 provides comparative results regarding the first eight natural frequencies corresponding to FG-GPLRC rectangular plates under the same GPL distribution patterns. As indicated by the data, the present results also exhibit good consistency with the corresponding reference results, with relatively small discrepancies for all considered modes. These comparisons further corroborate the efficacy of the developed framework for investigating the dynamic behavior associated with FG-GPLRC rectangular plates. Overall, the above validation results demonstrate that the unified modeling and solution framework developed in this work can accurately capture the free oscillation attributes of FG-GPLRC plate-shell composite systems, thereby providing a reliable theoretical foundation for subsequent dynamic analysis and stochastic vibration investigations of CPCS.

Table 4 and Table 5 present comparisons between the natural frequencies predicted by the present method and those obtained from finite element simulations for the CPCS and CPCS-DD under different GPL distribution patterns, respectively. It can be observed that, for both the CPCS without DVAs and the CPCS-DD with distributed DVAs, the results obtained by the present method show good agreement with the FEM solutions. As the mode order increases, slight discrepancies are observed in some higher-order modes; however, the maximum relative error is limited to 1.893%, which remains within an acceptable engineering tolerance. No significant numerical deviation or systematic error trend is observed. These comparative assessments convincingly validate the accuracy of the proposed formulation in reproducing the dynamic behavior associated with cylindrical shell–rectangular plate coupled structures, as well as those incorporating distributed DVAs. Therefore, the proposed approach provides a reliable theoretical foundation for the subsequent investigation of dynamic responses and vibration reduction performance under stochastic excitations.

**Figure 2:**
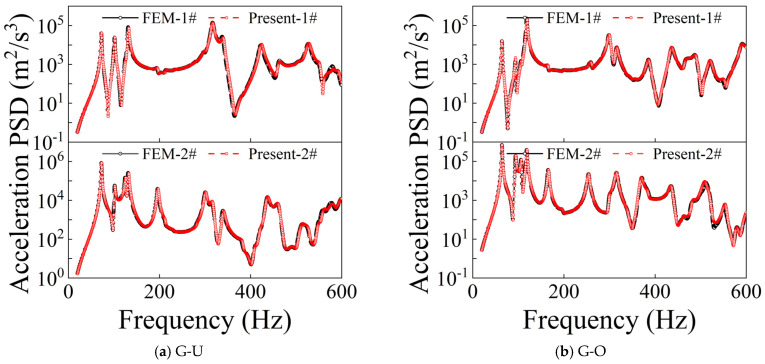

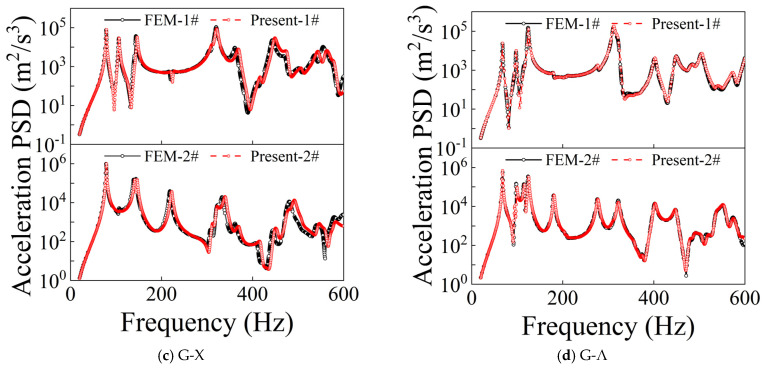
Comparison of random responses of CPCS.

**Figure 3:**
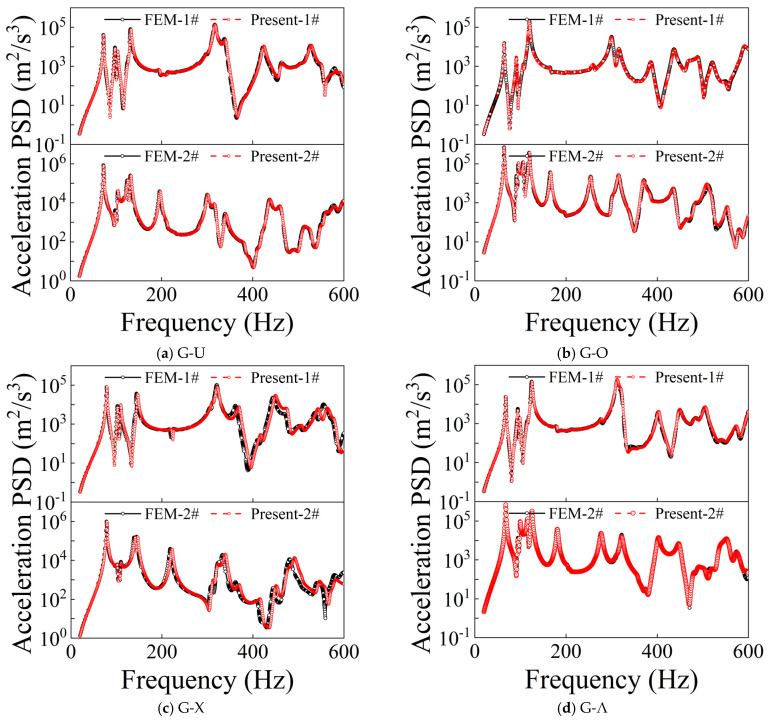
Comparison of random responses of CPCS-DD.

Figure 2 and Figure 3 present comparative results of the acceleration power spectral density (PSD) responses at two measurement points (1# and 2#) for CPCS and CPCS-DD structures under different GPL distribution types, subjected to random excitation and subjected to CC-CC boundary constraints. The comparison incorporates results derived from both the present approach and FEM calculation results. The results demonstrate that for all GPL distribution types, the random response curves obtained by the proposed method exhibit high consistency with FEM results in terms of overall amplitude levels, peak locations, and spectral distribution characteristics. The results demonstrate that the developed approach possesses good computational accuracy and reliability for investigating the dynamic response characteristics of the cylindrical shell and rectangular plate integrated system under random excitation, including when incorporating distributed DVAs.

### 3.2. Parametric Investigation

Building on the preceding analysis, this section conducts a parametric study to examine the dynamic performance of the integrated system under a range of scenarios, including different GPL distribution patterns, rectangular plate inclination angles, the thickness of both the rectangular plate and cylindrical shell, as well as the initial placement of the rectangular plate, and DVA parameters.

**Figure 4:**
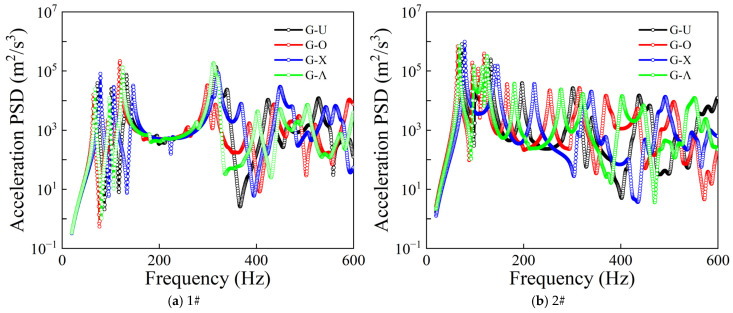
Comparison of PSD responses for different GPL distribution types.

Figure 4 presents the effects of different GPL distribution types on the acceleration PSD responses of the CPCS structure under random excitation, where Figure 4a,b correspond to measurement points 1# and 2#, respectively. It can be observed that the effect of GPL distribution patterns on the random response of the structure is mainly reflected in the amplitudes of resonance peaks, spectral distribution characteristics, and response levels in the high-frequency range. Within the scope of the analyzed profiles, the G-X pattern attains the maximum resonance frequency values when subjected to stationary stochastic loading, thereby implying that this specific configuration confers the superior global rigidity. Therefore, unless otherwise stated, the G-X GPL distribution is adopted in the subsequent analyses.

**Figure 5:**
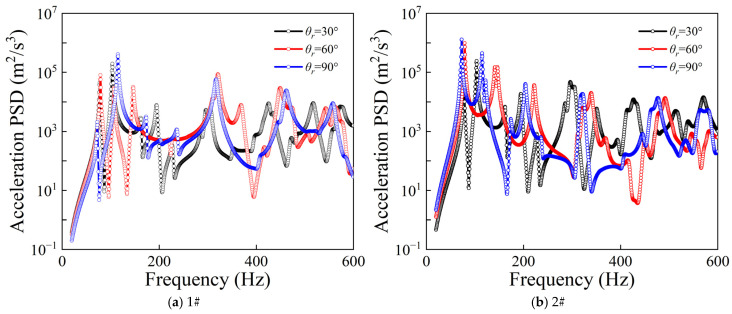
Comparison of PSD responses for different rectangular plate angles.

Figure 5 illustrates the influence of different rectangular plate angles *θ_r_* on the acceleration PSD responses of the CPCS structure under random excitation. It can be observed that, as the rectangular plate angle *θ_r_* increases, the overall dynamic characteristics of the structure change significantly, and the resonance peaks at various modes exhibit different degrees of frequency shift, indicating that the coupled stiffness and mass distribution of the plate–shell system are altered with the variation in *θ_r_*. In the low-frequency range, the differences in responses for different angles are relatively small, whereas in the mid- and high-frequency ranges, the separation of the PSD curves becomes more pronounced.

**Figure 6:**
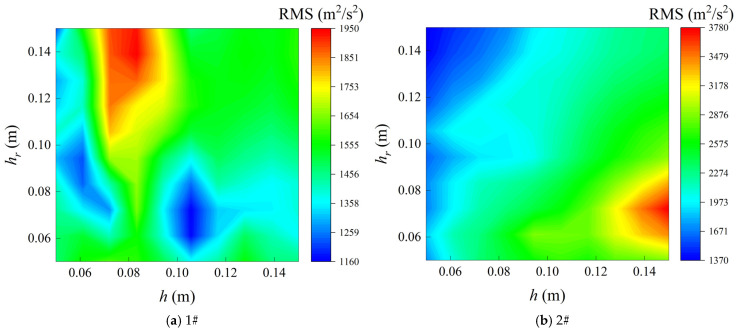
RMS contour maps for variations in cylindrical shell and rectangular plate thicknesses.

Figure 6 presents the contour maps of the acceleration root mean square (RMS) responses at two measurement points of the CPCS structure for different combinations of the cylindrical shell thickness *h* and the rectangular plate thickness *h_r_*. As shown in Figure 6a, the RMS response at measurement point 1# is more sensitive to variations in the cylindrical shell thickness *h*. When the cylindrical shell is relatively thin and the rectangular plate thickness is in a moderate-to-large range, the structural vibration response becomes pronounced, and the RMS value increases significantly. With increasing cylindrical shell thickness *h*, the overall response level exhibits a decreasing trend, indicating that increasing the shell thickness effectively enhances the structural stiffness. As illustrated in Figure 6b, the RMS response at measurement point 2# is more strongly influenced by changes in the rectangular plate thickness *h_r_*. When *h_r_* is small and the cylindrical shell thickness *h* is relatively large, the vibration response in the rectangular plate region reaches a higher level. As the rectangular plate thickness increases, the RMS response generally decreases, demonstrating that increasing the plate thickness is beneficial for reducing the vibration intensity of the plate structure under random excitation.

**Figure 7:**
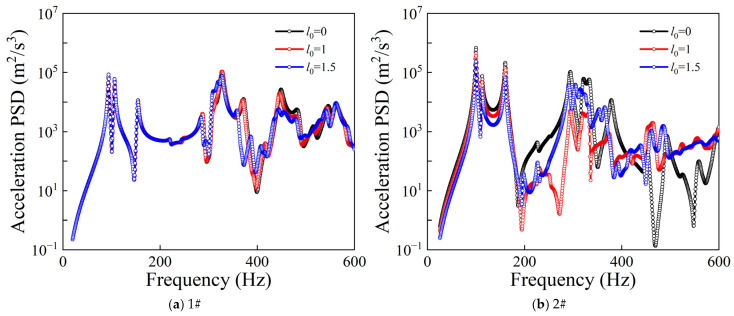
Comparison of PSD responses for different rectangular plate starting positions.

Figure 7 illustrates the influence of different rectangular plate starting positions *l*_0_ on the acceleration PSD responses of the CPCS. For measurement point 1#, the low-frequency responses under different *l*_0_ conditions show relatively small differences, and the locations of the dominant resonance peaks remain nearly unchanged. As the frequency increases, variations in the amplitudes of resonance peaks and local spectral distributions can be observed. In contrast, the response at measurement point 2# is more sensitive to changes in *l*_0_. In the mid- and high-frequency ranges, the PSD curves corresponding to different starting positions exhibit pronounced differences, and the resonance peak amplitudes at certain frequencies change significantly. This indicates that the starting position of the rectangular plate directly affects the equivalent stiffness distribution and local modal characteristics of the plate, thereby exerting a more direct modulation effect on the random vibration response.

**Figure 8:**
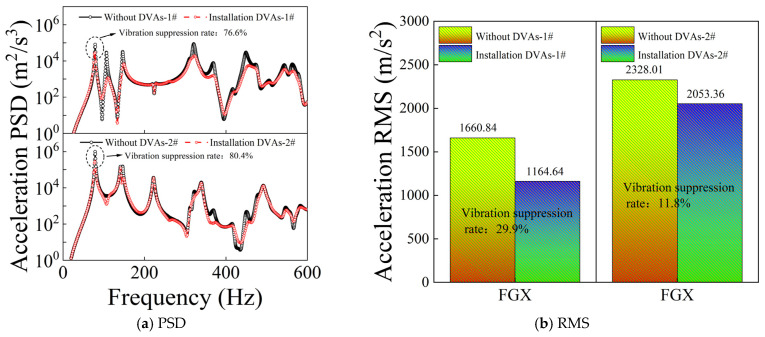
Comparison of PSD and RMS responses before and after installing DVAs.

In the parametric study of DVA characteristics, the mass was set to *m_d_* = 23 kg as a reference value. Figure 8 contrasts the stochastic response exhibited by the CPCS assembly in the absence and presence of DVAs. Figure 8a shows the acceleration PSD responses at the two measurement points, while Figure 8b illustrates the corresponding acceleration RMS results. As observed from Figure 8a, over the entire analyzed frequency range, the PSD curves at both measurement points are significantly lower after the installation of DVAs than those without DVAs. Specifically, at point 1#, the maximum PSD peak at approximately 79 Hz is reduced by 76.6% after installing the DVAs. At point 2#, an even more substantial peak reduction of 80.4% is achieved at the dominant resonance frequency around 79 Hz, demonstrating the high efficacy of the DVAs in mitigating resonant vibrations. Furthermore, the RMS comparison in Figure 8b quantifies the overall vibration reduction across the frequency spectrum. At point 1#, the overall RMS level is reduced by 29.9%, while at point 2#, a reduction of 11.8% is observed. These results collectively highlight the effectiveness of the proposed DVA implementation strategy in controlling vibrations.

**Figure 9:**
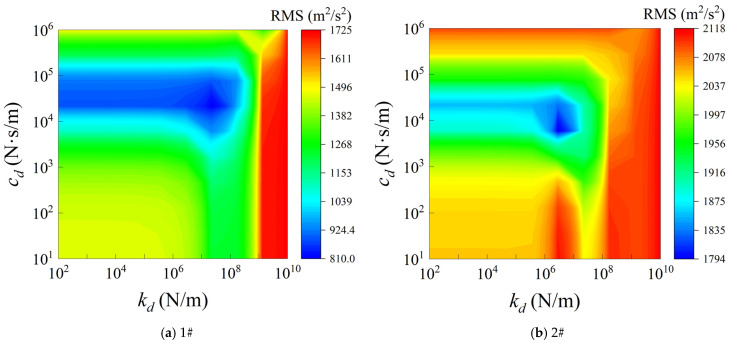
Effect of variations in DVAs stiffness and damping on RMS.

Figure 9 illustrates the effects of the DVAs stiffness and damping on the RMS responses of the structure. The results indicate that a relatively continuous and wide low-response region appears in the range of moderate damping combined with moderately high stiffness. As the DVAs stiffness or damping deviates from this optimal region, the random vibration response gradually increases. In particular, in the high-stiffness region, the RMS values increase significantly. This phenomenon indicates that excessively large stiffness introduces additional equivalent stiffness to the structure, which raises the local natural frequencies and weakens the tuning effectiveness of the DVAs, and may even lead to local vibration amplification. Moreover, both excessively large and excessively small damping reduce the energy dissipation efficiency. Therefore, within a proper combination range of stiffness and damping, the DVAs are able to efficiently redistribute and dissipate the vibrational energy of the system.

## 4. Conclusions

This study focuses on the dynamic response of cylindrical shell–rectangular plate coupled structures under random excitation, systematically investigating the effects of FG-GPLRC material variables, geometric dimensions of the structure, and distributed DVA arrays on the stochastic dynamic response of the structure. The main conclusions, validated through theoretical analysis and comparison with finite element results, are summarized as follows:

(1) The established stochastic dynamic model agrees well with the finite element results in terms of acceleration PSD and RMS responses, thereby verifying the precision and dependability of the developed method for examining the CPCS and its DVA-integrated configuration when subjected to random excitation.
(2) The type of GPL distribution significantly influences the stochastic response of the structure. Among them, the G-X type exhibits higher equivalent stiffness and superior dynamic performance, leading to more favorable response characteristics under random excitation.
(3) The inclination angle and initial position of the rectangular plate, along with the thicknesses of the cylindrical shell and rectangular plate, have substantial effects on the stochastic vibration response of the structure. These parameters primarily modulate the dynamic characteristics of the structure by altering the plate–shell coupling stiffness and mass distribution.
(4) Distributed DVAs can effectively reduce the PSD and RMS response levels of the CPCS under random excitation. The overall RMS values at points 1# and 2# are reduced by 29.9% and 11.8%, respectively, while the maximum PSD peaks are attenuated by 76.6% and 80.4%. These results confirm that properly designed DVAs enable effective vibration mitigation.
(5) This study highlights the significant advantages of the proposed dynamic model over traditional finite element methods in conducting parametric investigations. The model exhibits a considerably lower number of degrees of freedom than FEM, resulting in superior computational efficiency. Furthermore, the elimination of repetitive meshing during parameter adjustments further demonstrates the model’s utility as a practical tool for the design and optimization of complex coupled structures.

Despite these contributions, this study has several limitations. First, the findings are primarily based on theoretical analysis and numerical simulation and thus lack experimental validation to confirm the accuracy of the proposed model. Second, the current model is developed within the framework of linear vibration theory and does not incorporate the geometric or material nonlinearities that may become significant under strong excitation. Addressing these aspects constitutes a crucial direction for future research.

## Figures and Tables

**Table 1:** Convergence analysis of natural frequencies for the G-X type CPCS under CC-CC boundary conditions.

*M_xr_* × *N_y_*	*M_xc_*	Mode No.							
		1	2	3	4	5	6	7	8
5 × 5	4	92.71	114.67	129.01	129.56	158.34	164.18	176.94	219.05
	8	79.52	85.66	107.20	114.61	146.96	152.58	160.48	164.99
	12	79.35	85.38	106.76	114.05	146.77	152.52	159.49	163.56
	16	79.35	85.37	106.75	114.03	146.77	152.52	159.47	163.48
	20	79.35	85.37	106.75	114.03	146.77	152.52	159.47	163.48
10 × 10	4	90.24	112.54	128.47	128.88	129.33	157.36	161.80	169.06
	8	79.21	85.40	107.04	111.16	114.50	141.82	146.50	151.09
	12	79.02	85.08	106.60	111.04	113.93	141.61	146.31	150.50
	16	79.01	85.08	106.60	111.04	113.91	141.61	146.31	150.50
	20	79.01	85.08	106.60	111.04	113.91	141.61	146.31	150.50
15 × 15	4	90.18	112.48	128.44	128.87	129.33	157.33	161.76	169.00
	8	79.21	85.39	107.04	111.15	114.50	141.79	146.50	151.08
	12	79.01	85.08	106.60	111.03	113.91	141.55	146.30	150.49
	16	79.01	85.07	106.59	111.03	113.90	141.55	146.30	150.48
	20	79.01	85.07	106.59	111.03	113.90	141.55	146.30	150.48
20 × 20	4	90.18	112.47	128.44	128.87	129.33	157.33	161.76	169.00
	8	79.21	85.40	107.04	111.15	114.50	141.79	146.50	151.08
	12	79.01	85.08	106.60	111.03	113.91	141.55	146.30	150.49
	16	79.01	85.07	106.59	111.03	113.90	141.55	146.30	150.48
	20	79.01	85.07	106.59	111.03	113.90	141.55	146.30	150.48
FEM		78.25	84.41	106.03	109.23	113.71	138.96	143.58	149.32

**Table 2:** Comparison of natural frequencies of FG-GPLRC cylindrical shells.

Type	Theory	Mode No.							
		1	2	3	4	5	6	7	8
G-U	Present	176.53	182.86	223.52	232.85	311.80	313.02	332.35	333.67
	Ref. [46]	176.55	182.88	223.54	232.87	311.81	313.08	332.39	333.68
	Different (%)	−0.0118	−0.0117	−0.0067	−0.0086	−0.0064	−0.0180	−0.0121	−0.0016
G-O	Present	158.93	166.45	190.96	219.98	250.34	281.18	288.38	314.39
	Ref. [46]	158.96	166.48	190.98	220.00	250.36	281.26	288.47	314.44
	Different (%)	−0.0177	−0.0170	−0.0127	−0.0094	−0.0089	−0.0262	−0.0293	−0.0168
G-X	Present	185.73	203.46	226.80	267.34	334.90	340.87	348.65	361.42
	Ref. [46]	185.75	203.48	226.82	267.36	334.90	340.92	348.69	361.44
	Different (%)	−0.0092	−0.0089	−0.0053	−0.0067	−0.0013	−0.0142	−0.0098	−0.0051
G-Λ	Present	166.21	168.93	204.50	220.30	270.52	290.64	304.43	319.03
	Ref. [46]	166.24	168.95	204.52	220.32	270.54	290.71	304.50	319.08
	Different (%)	−0.0147	−0.0145	−0.0107	−0.0081	−0.0077	−0.0222	−0.0246	−0.0145

**Table 3:** Comparison of natural frequencies of FG-GPLRC rectangular plates.

Type	Theory	Mode No.							
		1	2	3	4	5	6	7	8
G-U	Present	198.96	297.60	376.92	502.56	502.56	877.94	883.83	883.83
	Ref. [40]	198.85	297.40	376.68	502.38	502.38	877.86	883.29	883.29
	Different (%)	0.0550	0.0660	0.0634	0.0355	0.0355	0.0088	0.0612	0.0613
G-O	Present	167.06	248.57	315.74	423.95	423.95	747.14	748.14	748.14
	Ref. [40]	167.00	248.41	315.55	423.93	423.93	747.46	747.72	747.72
	Different (%)	0.0370	0.0644	0.0589	0.0048	0.0048	−0.0429	0.0563	0.0563
G-X	Present	225.73	339.06	428.14	567.57	567.58	983.61	994.03	994.04
	Ref. [40]	225.59	338.83	427.86	567.29	567.29	983.27	993.39	993.39
	Different (%)	0.0618	0.0677	0.0666	0.0500	0.0502	0.0341	0.0649	0.0658
G-Λ	Present	182.96	272.46	345.66	462.22	462.22	809.86	813.24	813.24
	Ref. [40]	182.88	272.29	345.45	462.12	462.12	809.98	812.77	812.77
	Different (%)	0.0463	0.0619	0.0598	0.0221	0.0222	−0.0149	0.0578	0.0582

**Table 4:** Natural frequency comparison for the CPCS.

Type	Theory	Mode No.							
		1	2	3	4	5	6	7	8
G-U	Present	72.889	80.40	100.30	101.67	113.54	126.17	132.45	135.28
	FEM	72.47	80.02	99.31	101.36	113.43	124.96	131.15	134.91
	Different (%)	0.5782	0.4711	0.9949	0.3058	0.0987	0.9707	0.9943	0.2720
G-O	Present	64.443	75.168	86.50	94.21	107.09	112.29	115.43	118.97
	FEM	64.08	74.91	85.67	93.77	106.17	112.19	115.27	118.34
	Different (%)	0.5649	0.3391	0.9701	0.4746	0.8684	0.0874	0.1362	0.5332
G-X	Present	79.008	85.07	106.59	111.03	113.90	141.55	146.30	150.48
	FEM	78.25	84.41	106.03	109.23	113.71	138.96	143.58	149.32
	Different (%)	0.9751	0.7748	0.5263	1.6470	0.1645	1.8624	1.8930	0.7735
G-Λ	Present	67.943	77.04	92.82	97.61	113.38	116.08	122.93	124.59
	FEM	67.67	77.02	91.94	97.57	113.77	114.93	122.65	123.92
	Different (%)	0.4094	0.0234	0.9637	0.0410	−0.3454	0.9997	0.2275	0.5366

**Table 5:** Natural frequency comparison for the CPCS-DD.

Type	Theory	Mode No.							
		1	2	3	15	28	29	30	31
G-U	Present	72.826	79.192	97.887	100.439	101.329	103.986	114.584	126.205
	FEM	72.409	78.838	97.737	100.34	101.19	103.71	114.47	124.99
	Different (%)	0.576	0.449	0.153	0.099	0.137	0.266	0.100	0.972
G-O	Present	64.409	74.036	86.391	93.059	93.436	95.433	107.145	113.128
	FEM	64.049	73.799	85.578	92.976	93.323	95.065	106.22	113.04
	Different (%)	0.562	0.321	0.950	0.089	0.121	0.387	0.871	0.078
G-X	Present	78.892	83.738	102.422	105.312	109.438	111.208	115.498	141.572
	FEM	78.138	83.126	102.11	105.17	109.01	109.46	115.31	138.98
	Different (%)	0.965	0.736	0.306	0.135	0.393	1.597	0.163	1.865
G-Λ	Present	67.897	75.908	92.601	96.417	97.002	99.332	114.154	116.124
	FEM	67.622	75.893	91.768	96.322	96.886	99.187	114.54	115.98
	Different (%)	0.407	0.020	0.908	0.099	0.120	0.146	−0.337	0.124

## Data Availability

The original contributions presented in this study are included in the article. Further inquiries can be directed to the corresponding author.
